# Supplementary material for: Identifying regulators of associative learning using a protein-labelling approach in Caenorhabditis elegans
Source: eLife. 2026 Jan 28;14:RP108438. doi: 10.7554/eLife.108438 (PMC12851583; doi:10.7554/eLife.108438)
Supplement: Figure 1—source data 1. [file elife-108438-fig1-data1.zip › Figure 1C-source data 1/Figure 1C-source data 1.pdf]

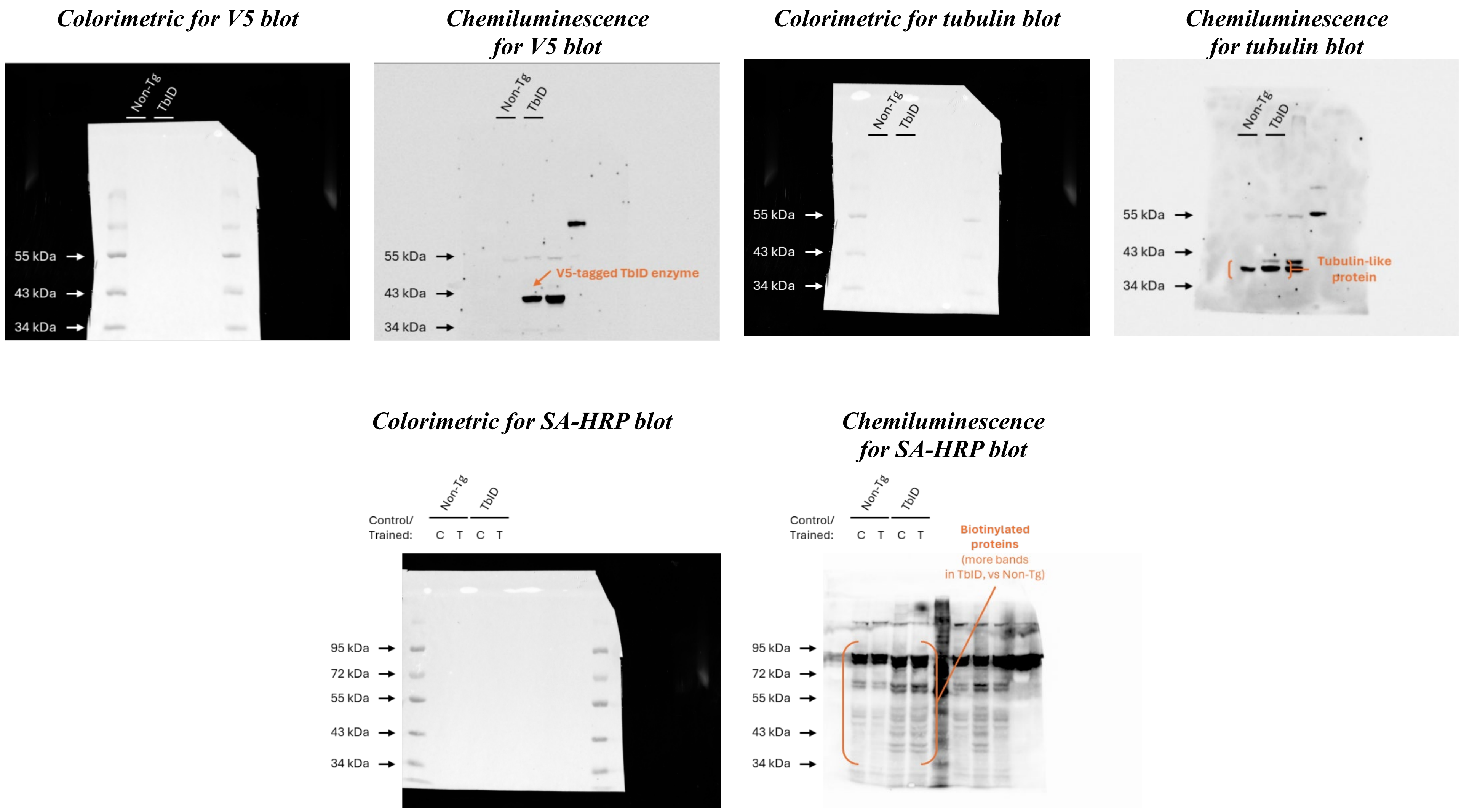

Figure 1C, Source Data 1. Original membrane images corresponding to **Figure 1C**. Membranes in the top row correspond to **Figure 1C, left panel** using (A) antibodies to visualise V5-tagged TurboID enzyme (in TurboID/TbID worms, versus non-transgenic/Non-Tg animals) and (B) antibodies for tubulin-like protein (as a loading control). The bottom row includes **Figure 1C, right panel** images using streptavidin-horseradish peroxidase (SA-HRP) to visualise biotinylated proteins. These proteins were all imaged by chemiluminescence. *C. elegans* lines (TbID vs Non-Tg) and treatment strategies are

annotated on the top of each image. Animals used to generate **Figure 1C, right panel** images were high-salt control (C) or trained (T) by salt associative learning. Leftmost lanes contain protein ladder (shown in ‘colorimetric’ images). Molecular weights for protein ladder standards are annotated on the left side of each image (in kDa). Relevant bands are annotated in orange.
