# Supplementary figures and images for: Identifying regulators of associative learning using a protein-labelling approach in Caenorhabditis elegans
Source: eLife. 2026 Jan 28;14:RP108438. doi: 10.7554/eLife.108438 (PMC12851583; doi:10.7554/eLife.108438)

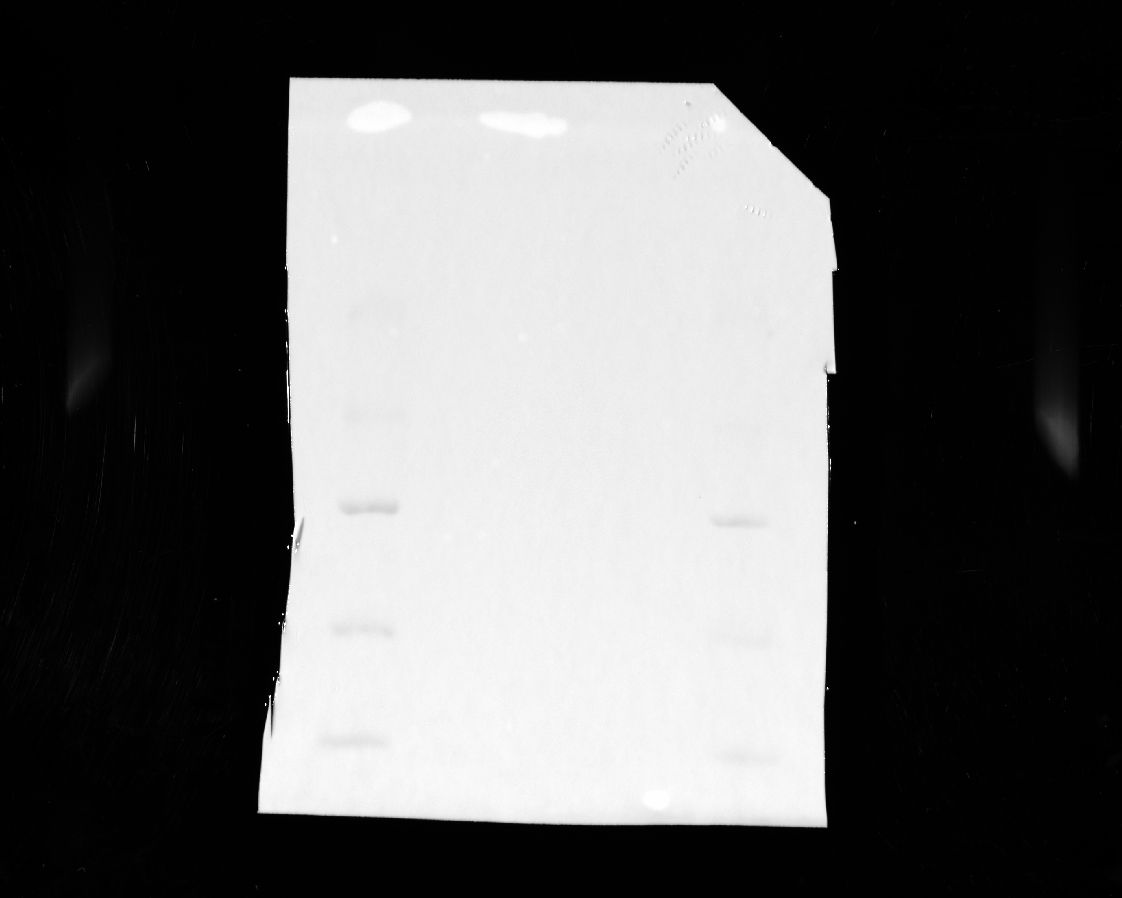

Supplement: Figure 1—source data 2. [file elife-108438-fig1-data2.zip › Figure 1C-source data 2/Figure 1C (left panel - ladder, for tubulin).tif]

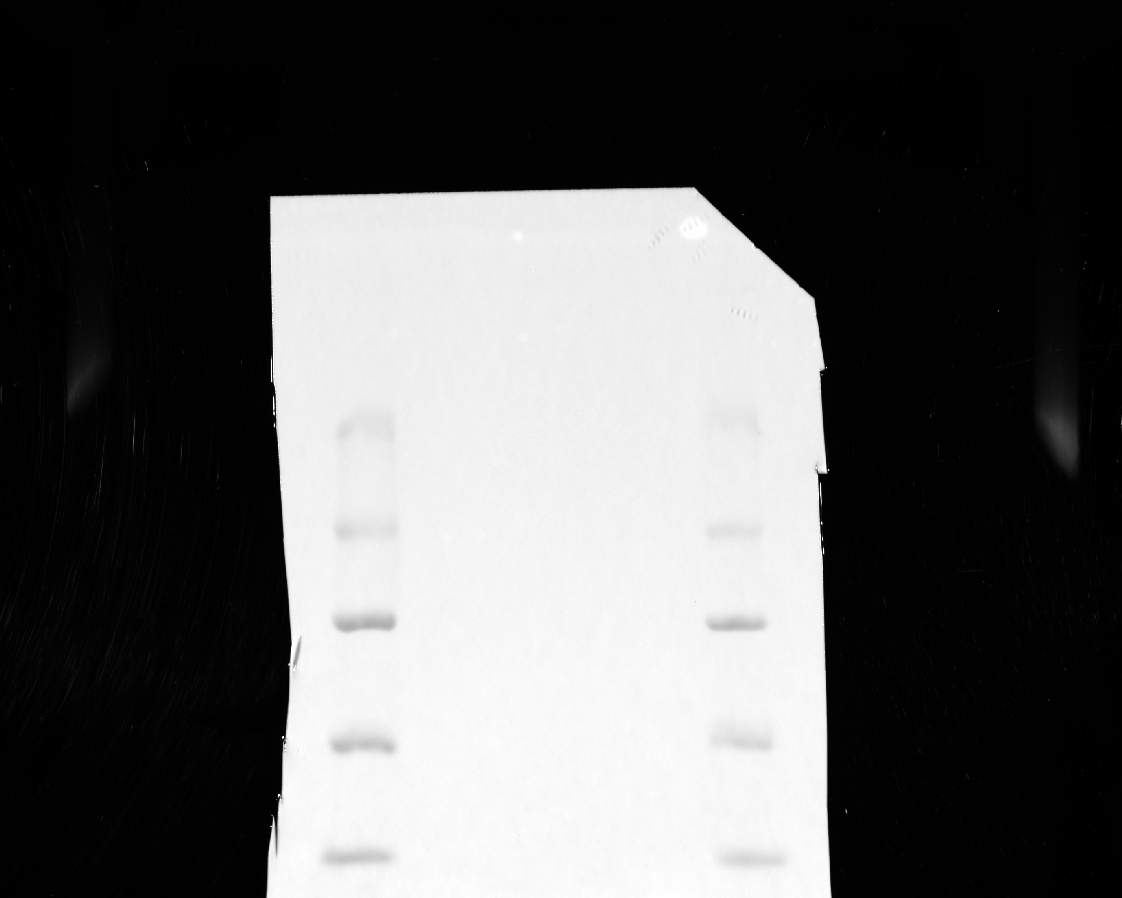

Supplement: Figure 1—source data 2. [file elife-108438-fig1-data2.zip › Figure 1C-source data 2/Figure 1C (left panel - ladder, for V5).tif]

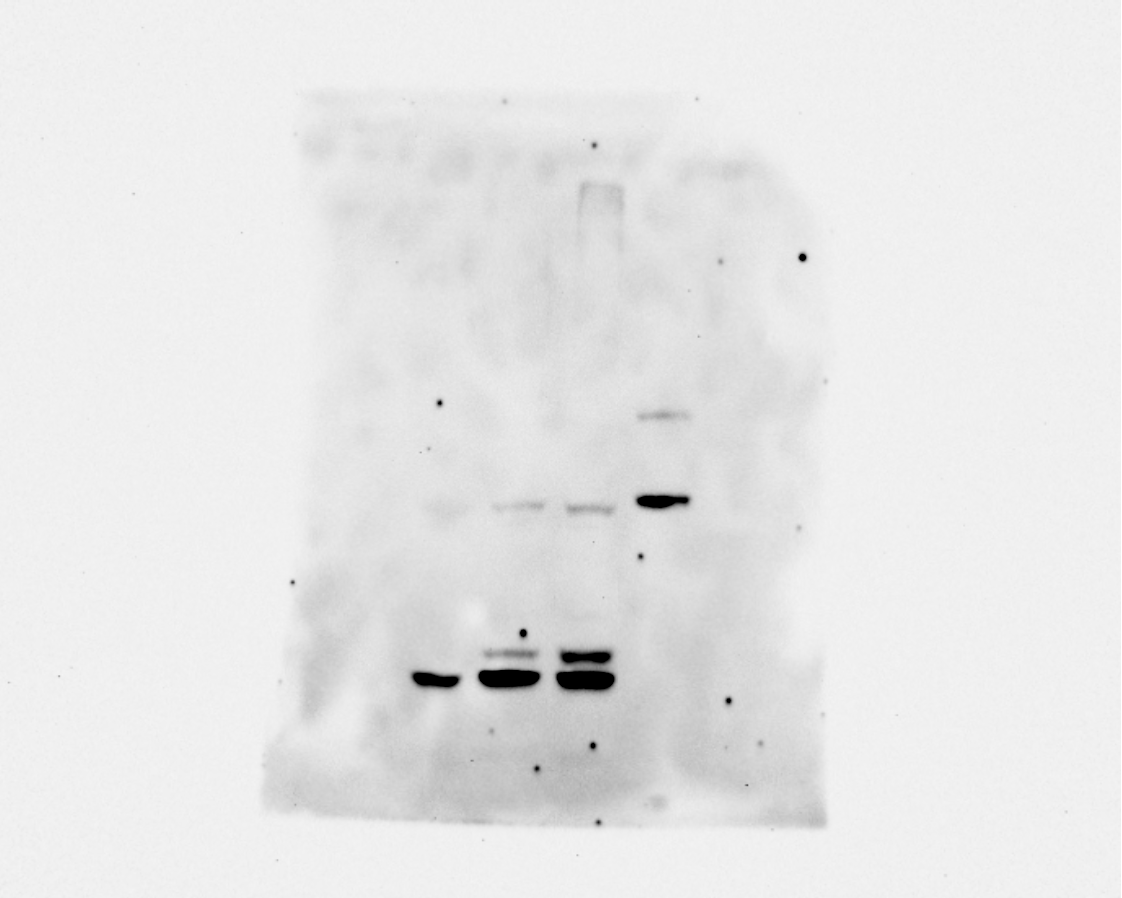

Supplement: Figure 1—source data 2. [file elife-108438-fig1-data2.zip › Figure 1C-source data 2/Figure 1C (left panel - tubulin).tif]

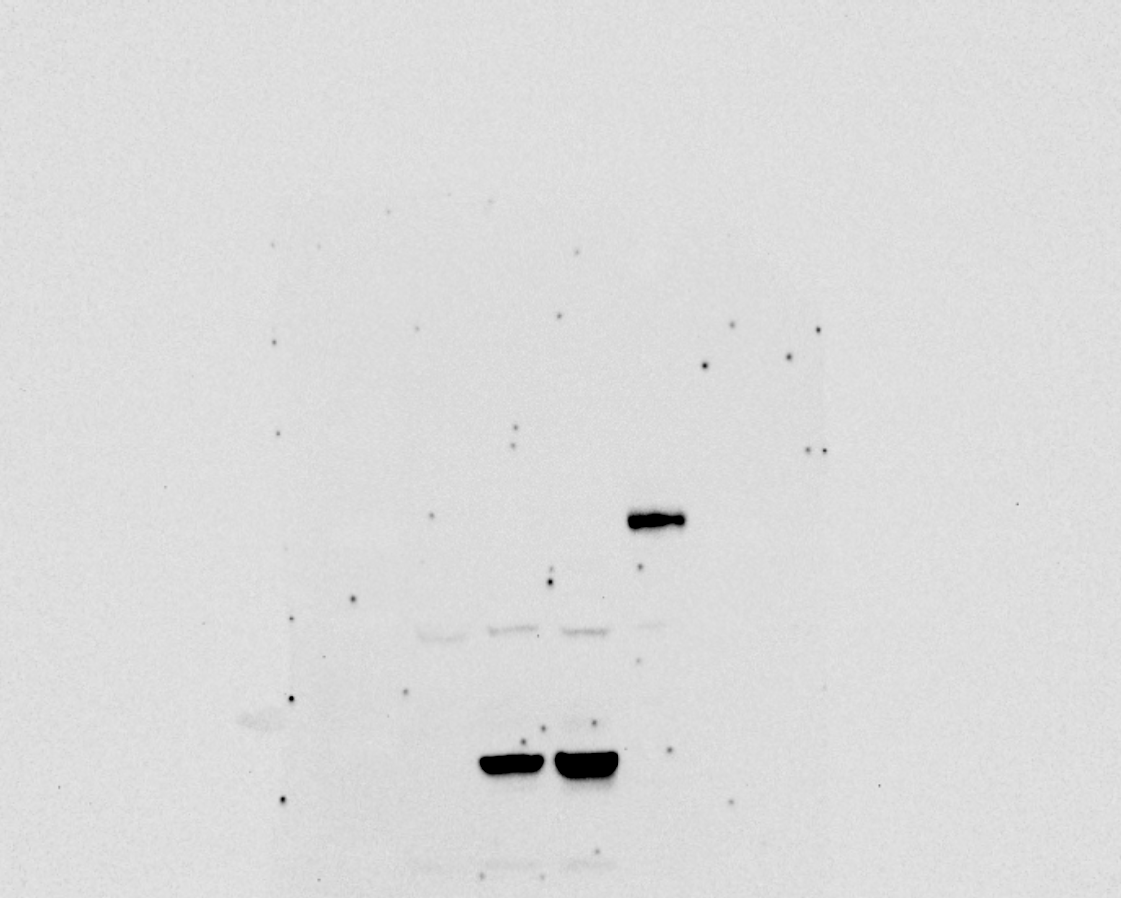

Supplement: Figure 1—source data 2. [file elife-108438-fig1-data2.zip › Figure 1C-source data 2/Figure 1C (left panel - V5).tif]

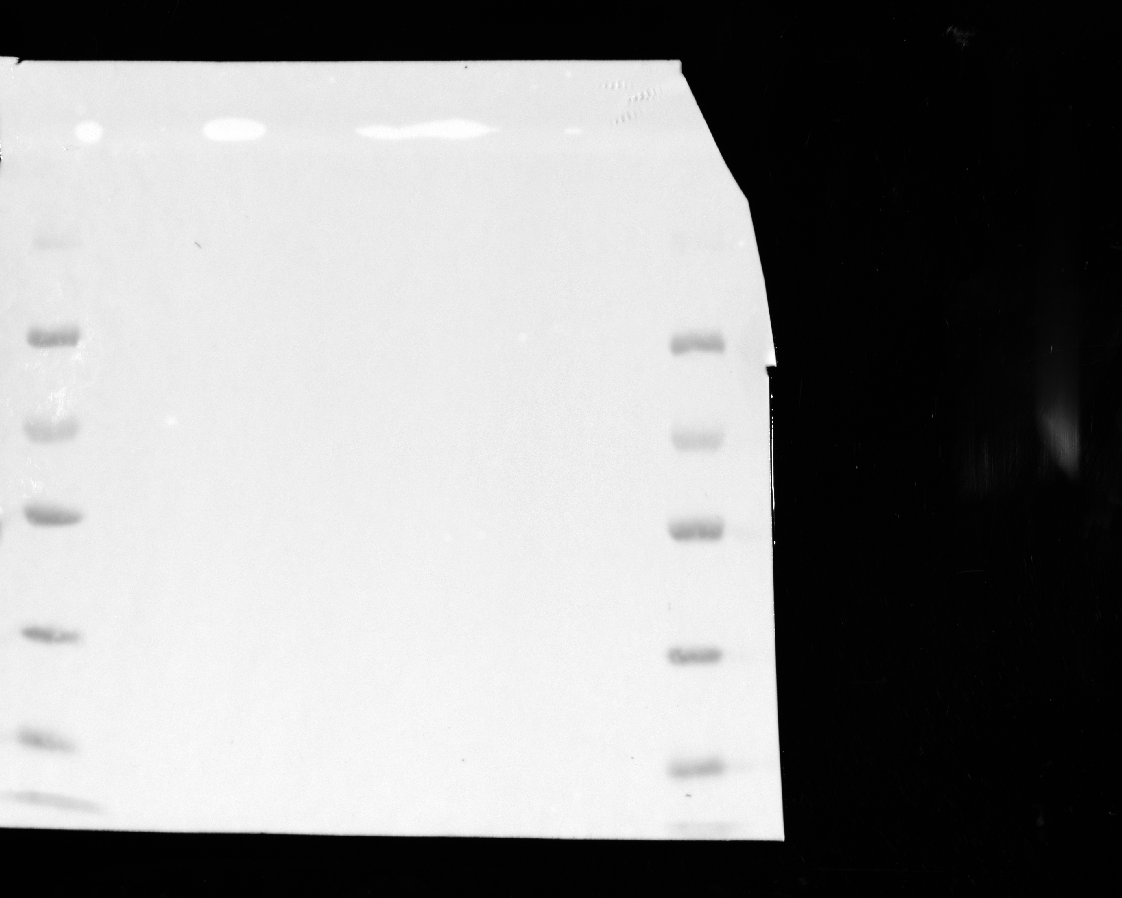

Supplement: Figure 1—source data 2. [file elife-108438-fig1-data2.zip › Figure 1C-source data 2/Figure 1C (right panel - ladder).tif]

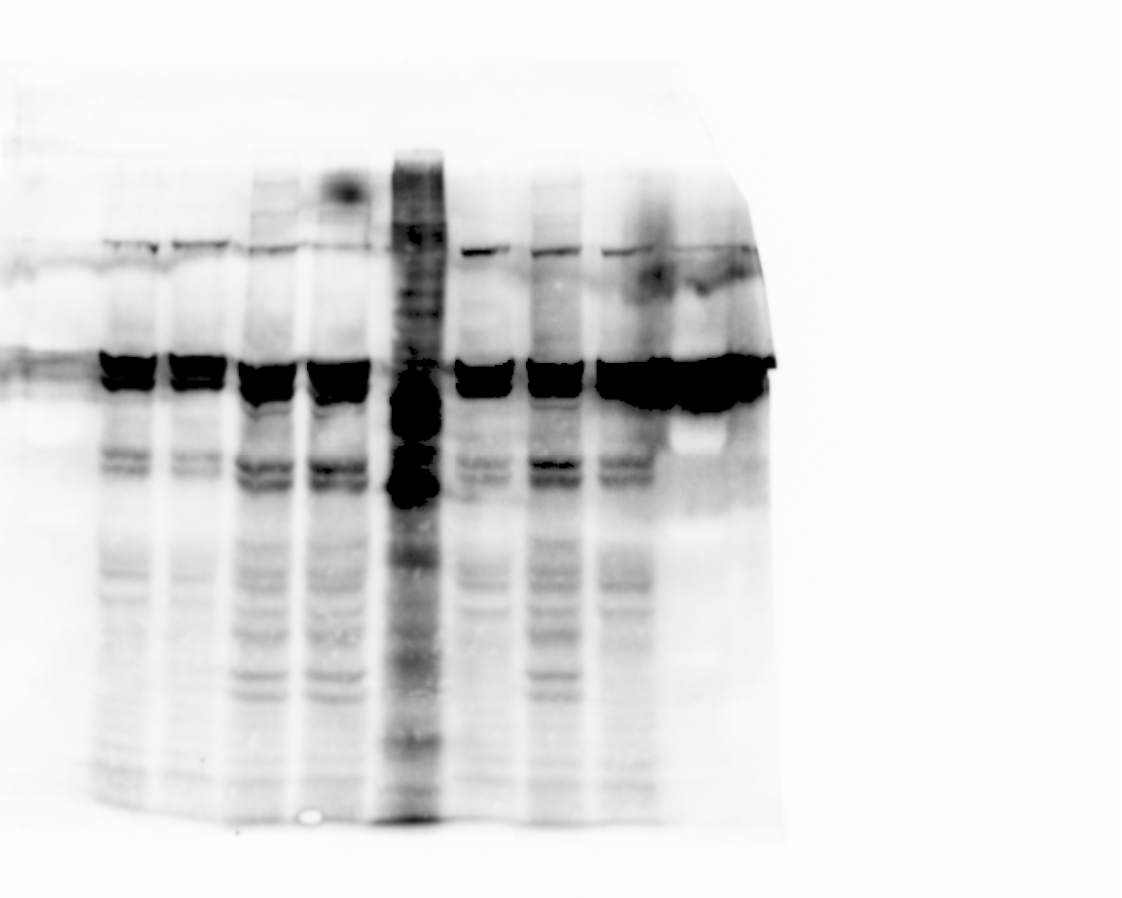

Supplement: Figure 1—source data 2. [file elife-108438-fig1-data2.zip › Figure 1C-source data 2/Figure 1C (right panel - SA-HRP).tif]

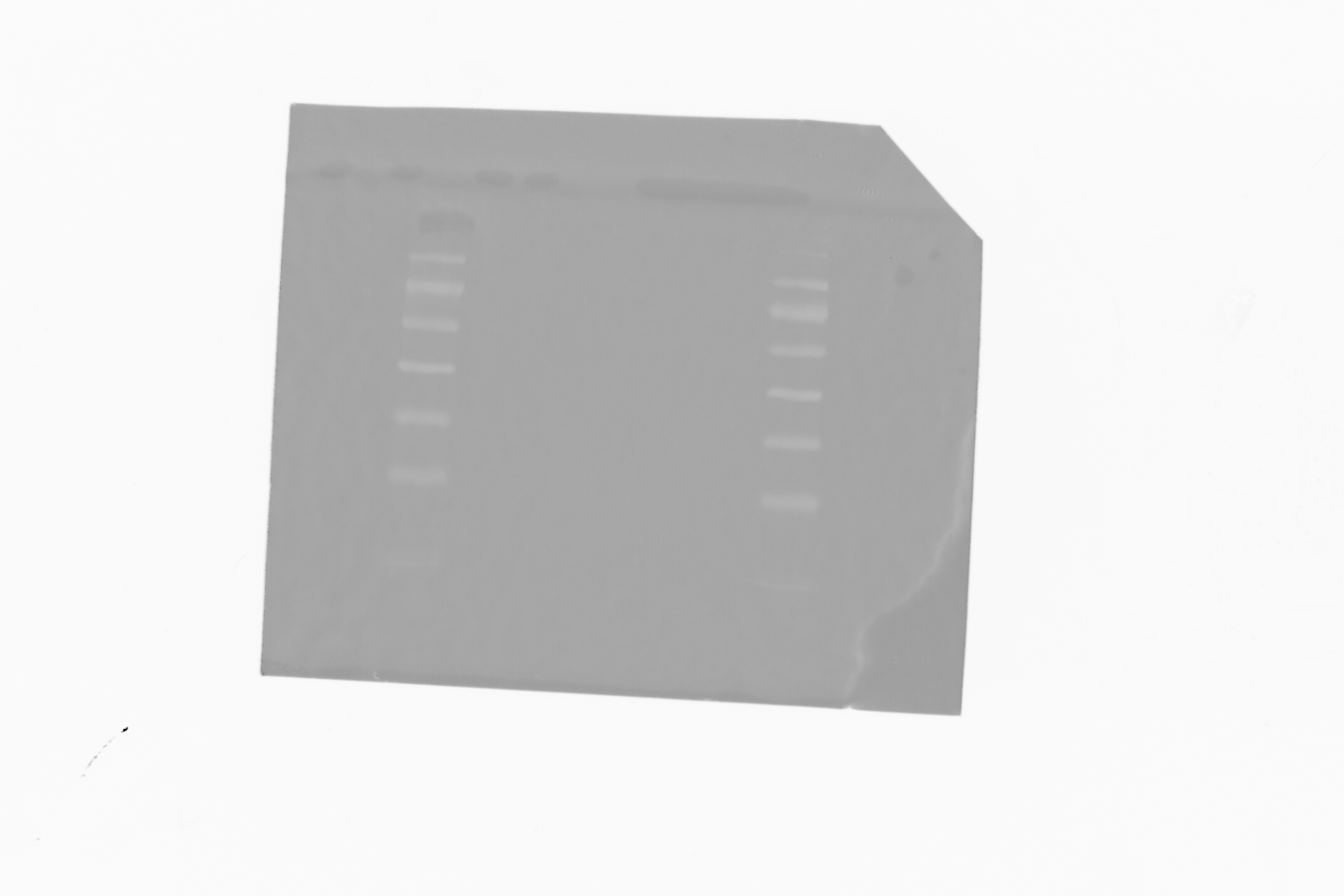

Supplement: Figure 1—figure supplement 1—source data 2. [file elife-108438-fig1-figsupp1-data2.zip › Figure 1-figure supplement 1-source data 2/Figure 1-figure supplement 1 (ladder).gel]

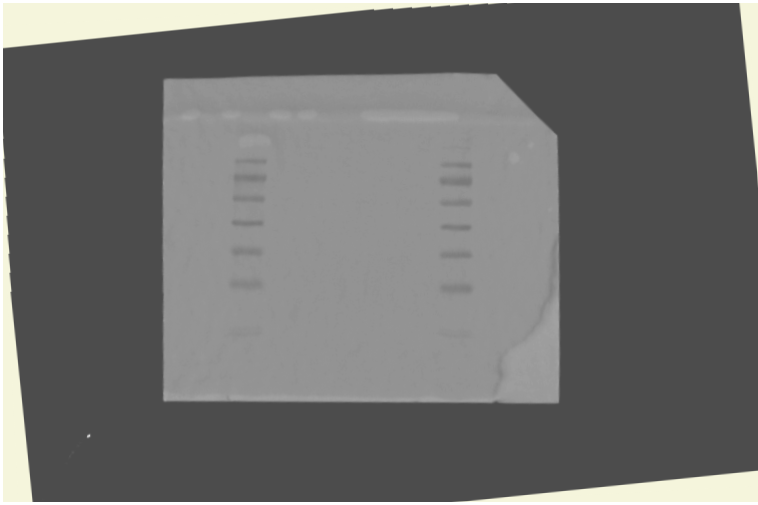

Supplement: Figure 1—figure supplement 1—source data 2. [file elife-108438-fig1-figsupp1-data2.zip › Figure 1-figure supplement 1-source data 2/Figure 1-figure supplement 1 (ladder).png]

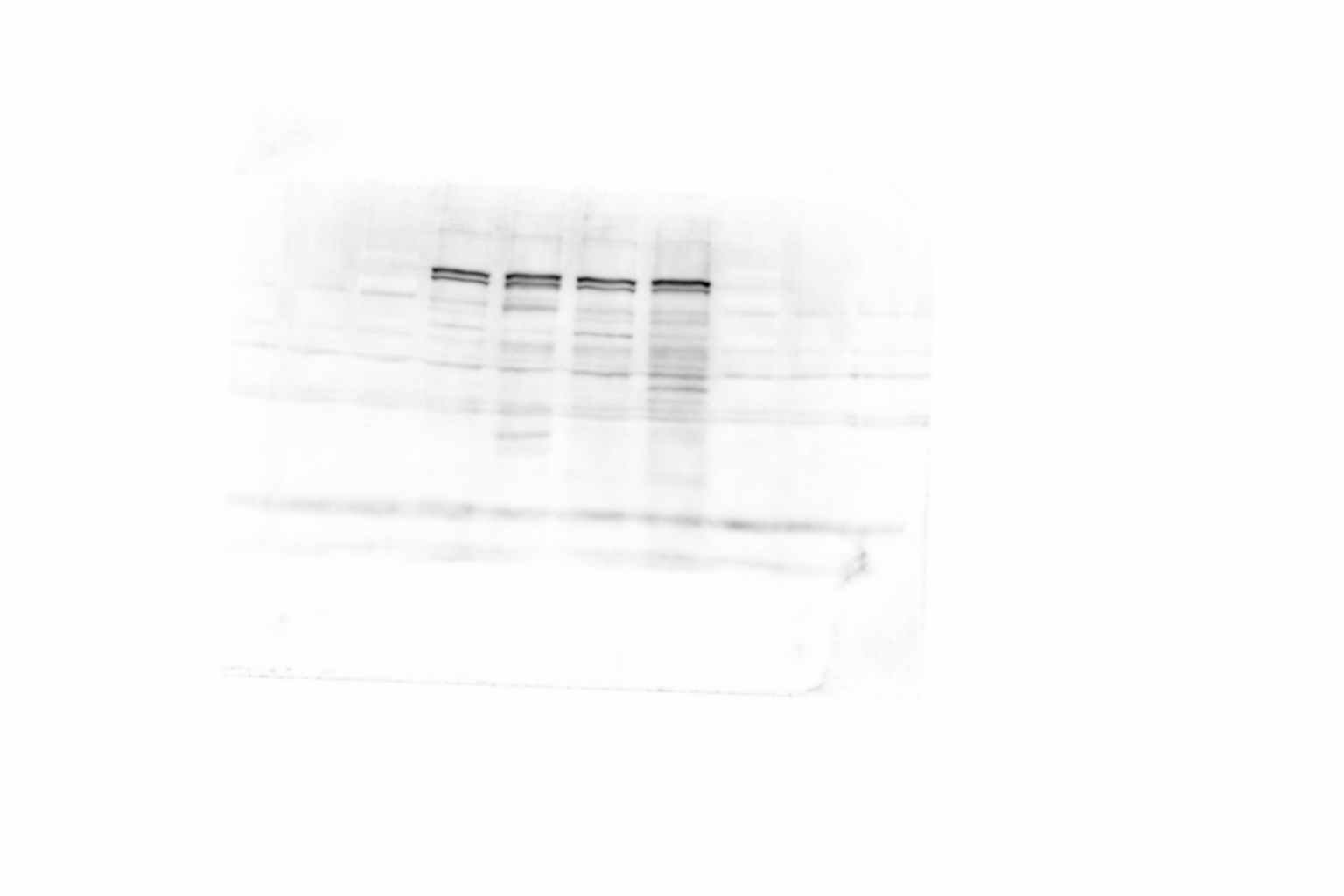

Supplement: Figure 1—figure supplement 1—source data 2. [file elife-108438-fig1-figsupp1-data2.zip › Figure 1-figure supplement 1-source data 2/Figure 1-figure supplement 1 (SA-HRP).gel]

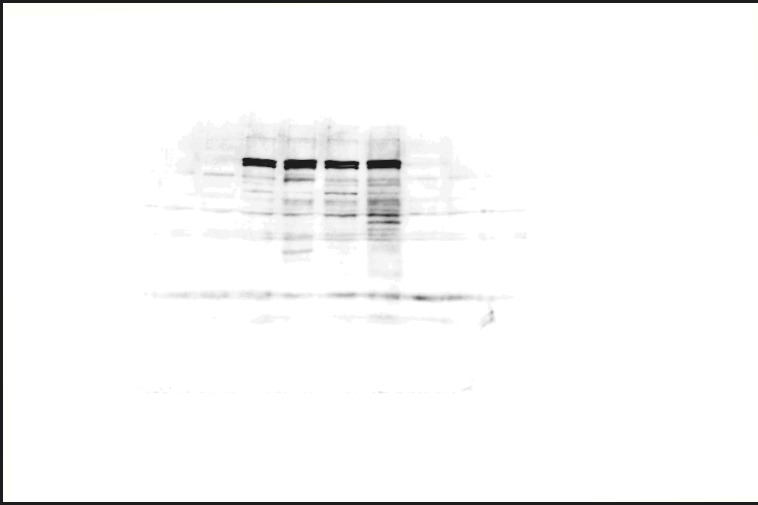

Supplement: Figure 1—figure supplement 1—source data 2. [file elife-108438-fig1-figsupp1-data2.zip › Figure 1-figure supplement 1-source data 2/Figure 1-figure supplement 1 (SA-HRP).png]

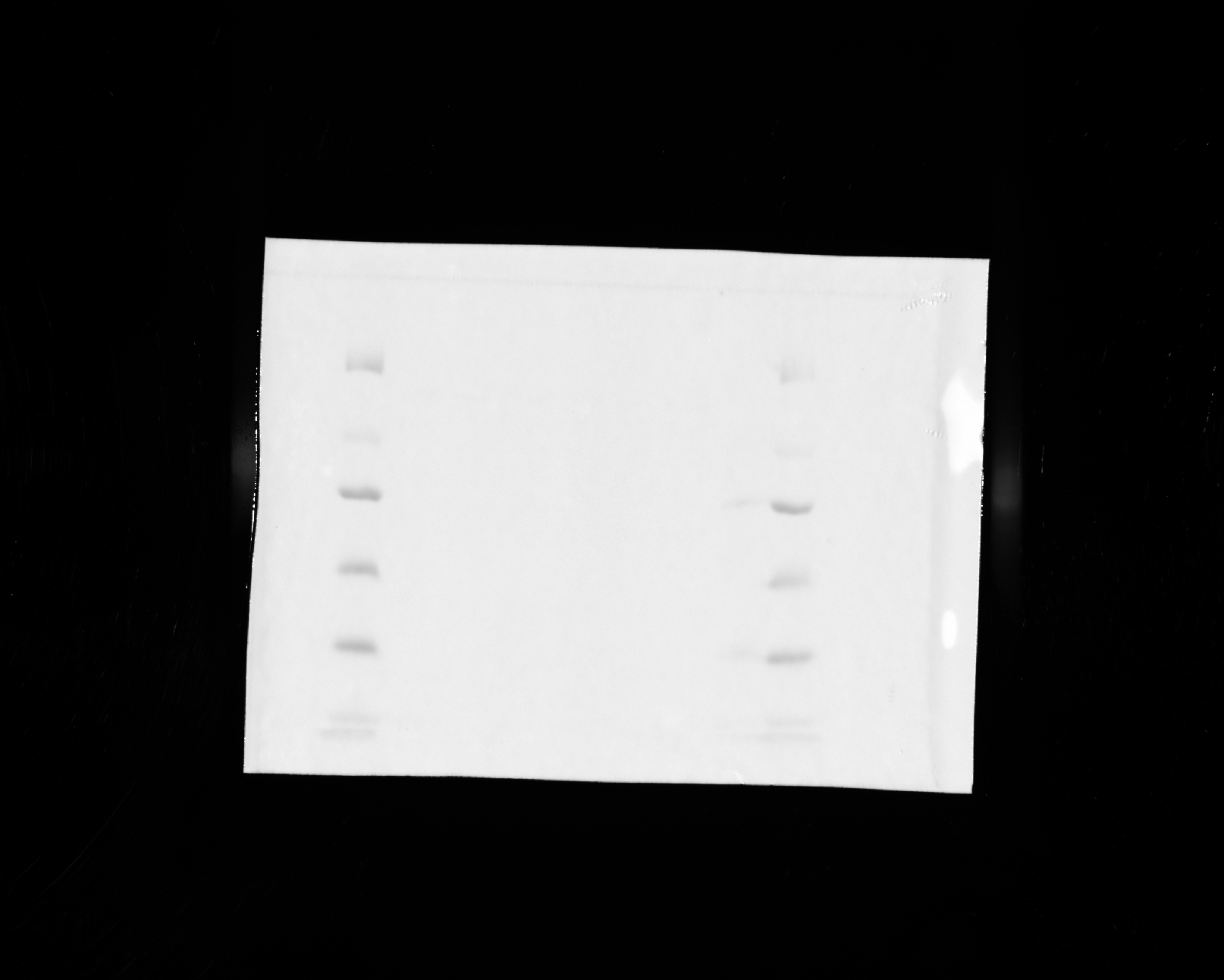

Supplement: Figure 1—figure supplement 3—source data 2. [file elife-108438-fig1-figsupp3-data2.zip › Figure 1-figure supplement 3-source data 2/Figure 1-figure supplement 3A (ladder).tif]

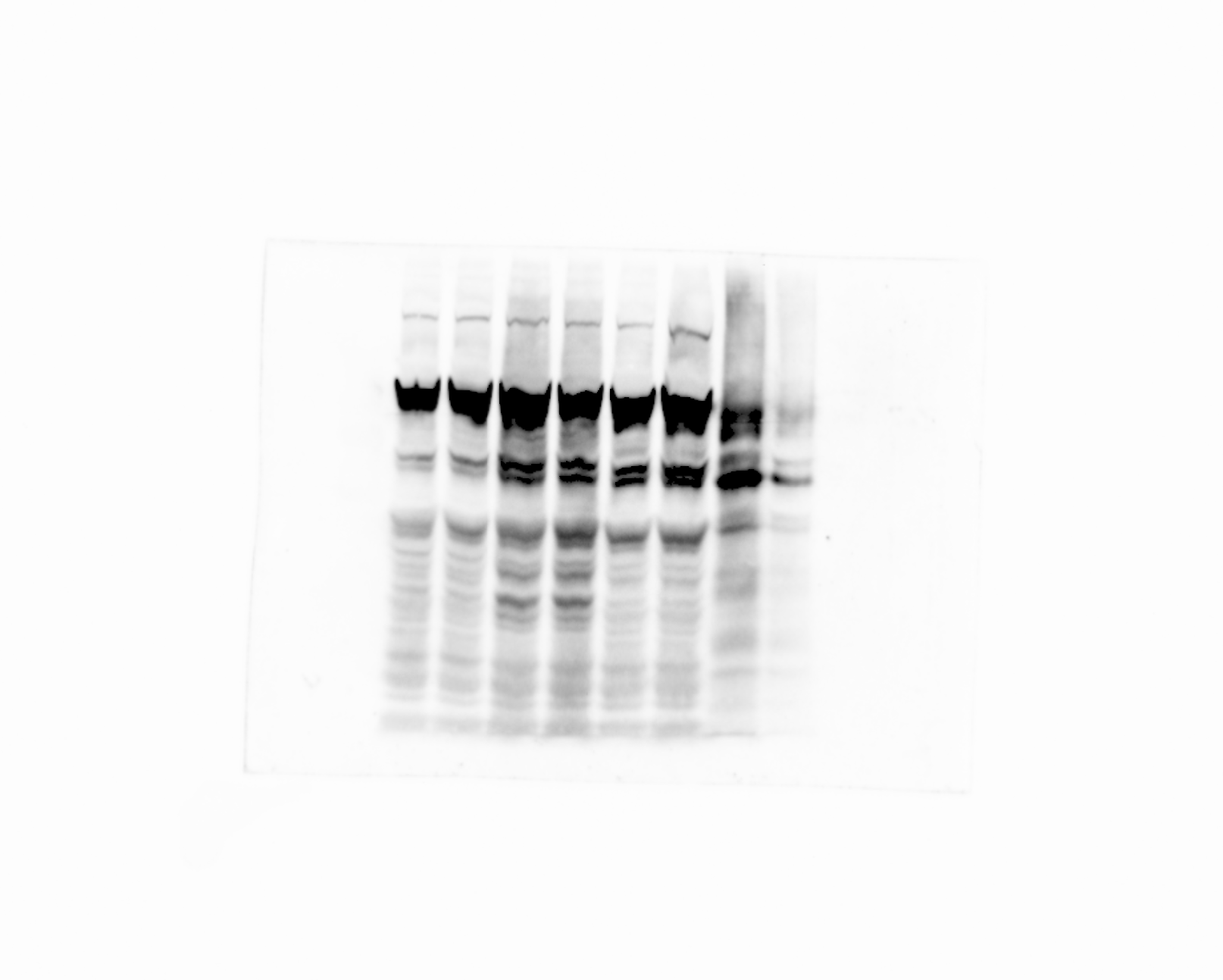

Supplement: Figure 1—figure supplement 3—source data 2. [file elife-108438-fig1-figsupp3-data2.zip › Figure 1-figure supplement 3-source data 2/Figure 1-figure supplement 3A (SA-HRP).tif]

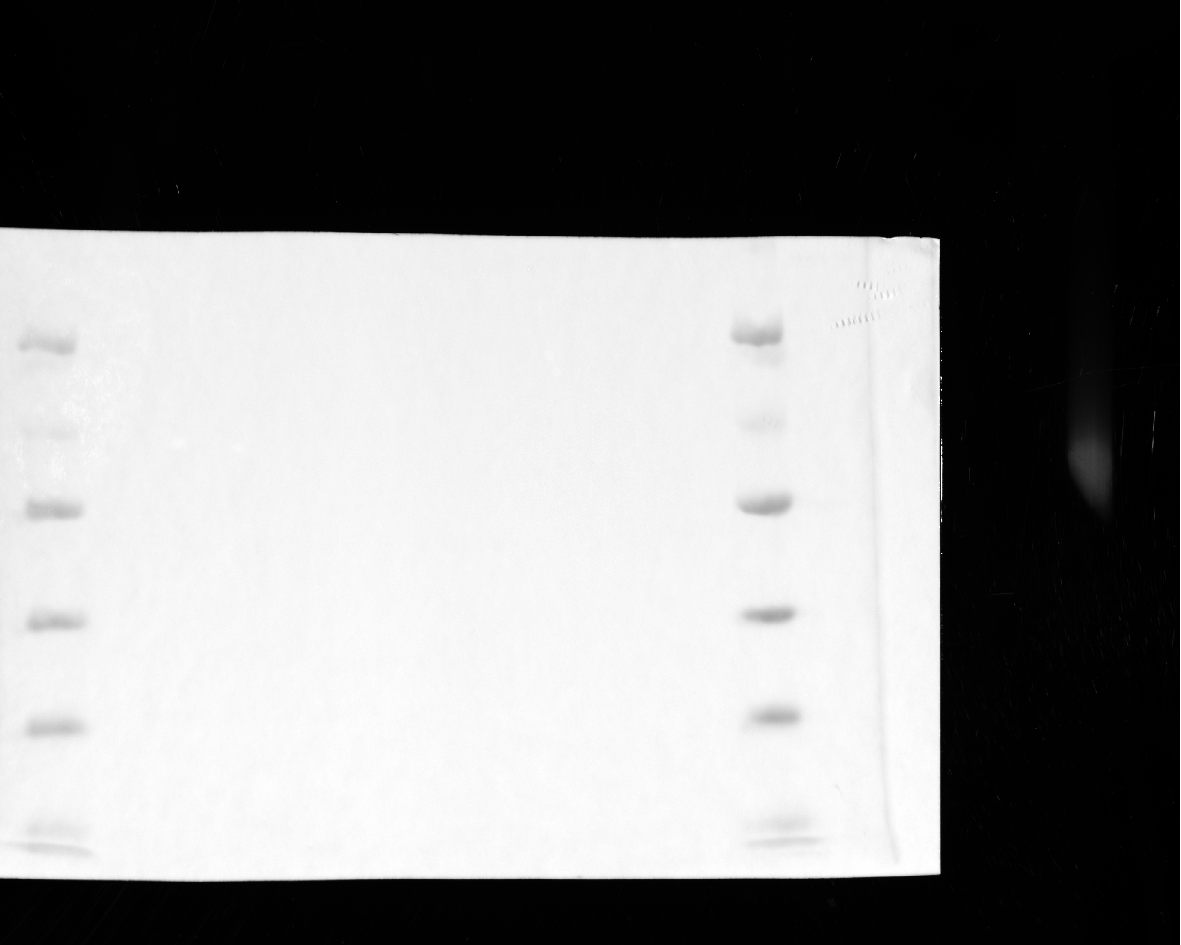

Supplement: Figure 1—figure supplement 3—source data 2. [file elife-108438-fig1-figsupp3-data2.zip › Figure 1-figure supplement 3-source data 2/Figure 1-figure supplement 3B (ladder).tif]

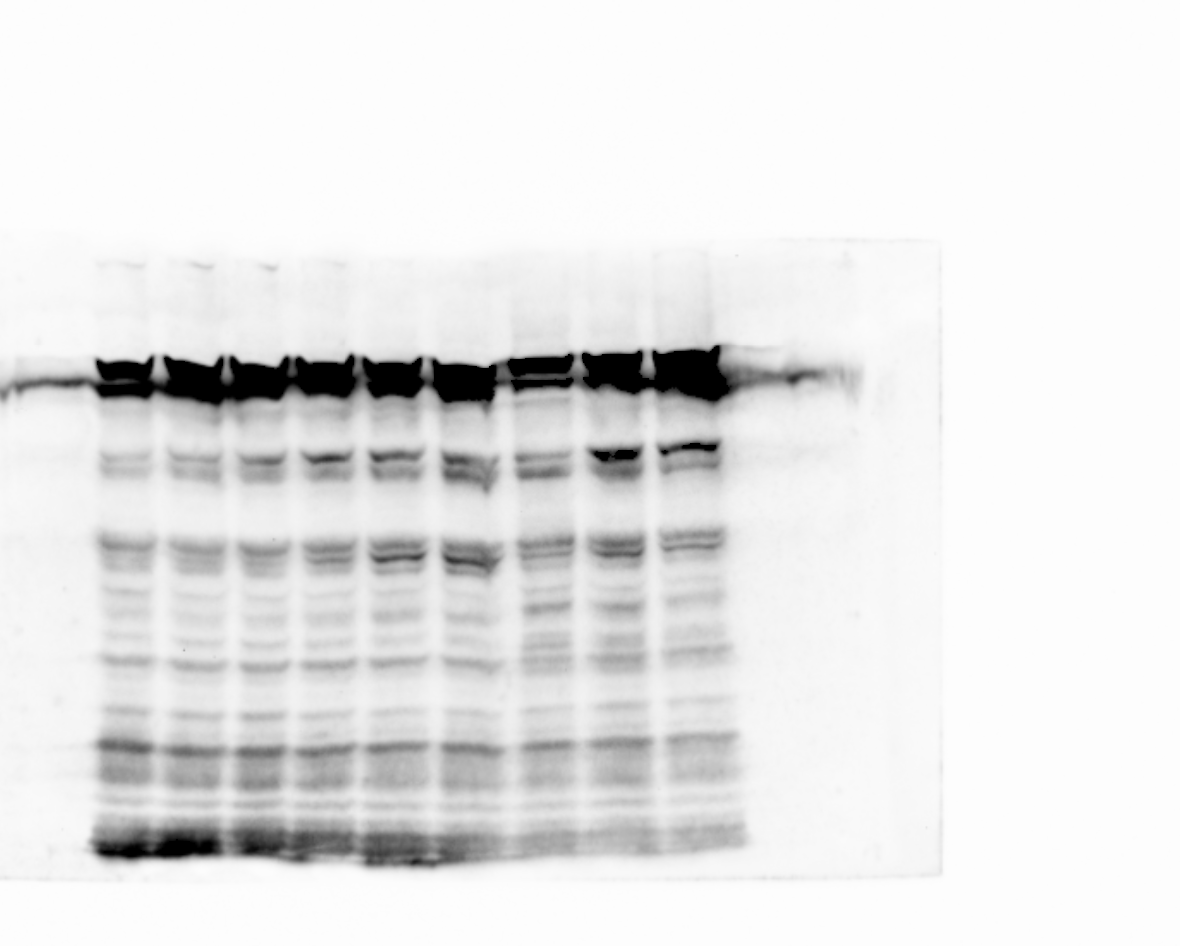

Supplement: Figure 1—figure supplement 3—source data 2. [file elife-108438-fig1-figsupp3-data2.zip › Figure 1-figure supplement 3-source data 2/Figure 1-figure supplement 3B (SA-HRP).tif]

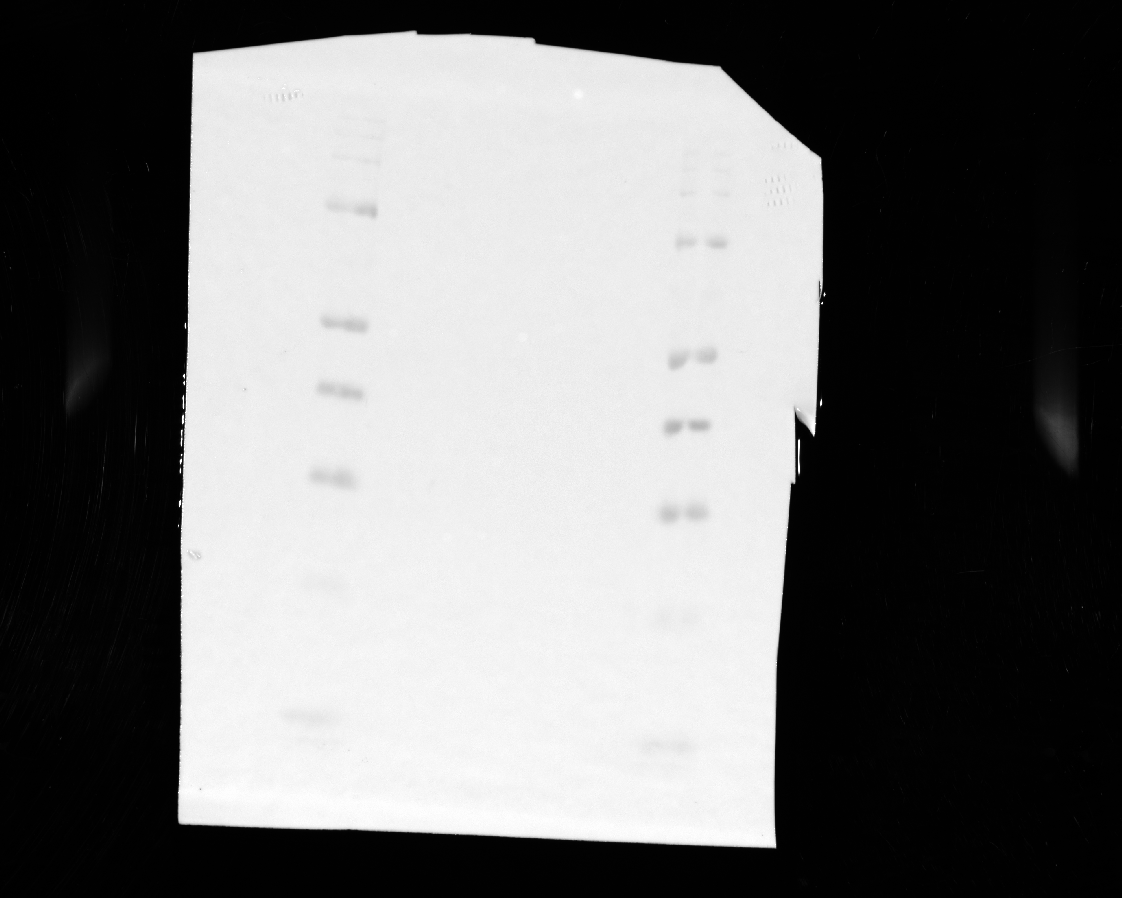

Supplement: Figure 1—figure supplement 3—source data 2. [file elife-108438-fig1-figsupp3-data2.zip › Figure 1-figure supplement 3-source data 2/Figure 1-figure supplement 3C (ladder).tif]

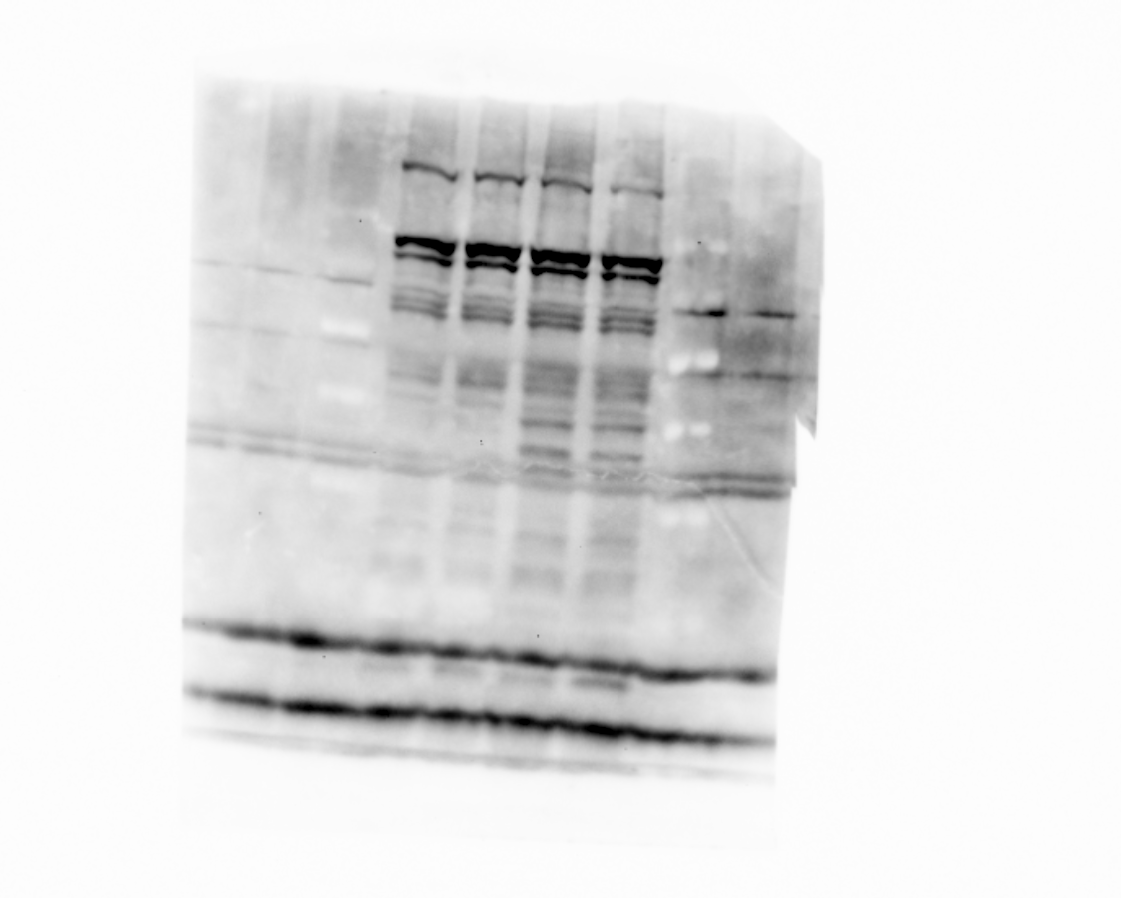

Supplement: Figure 1—figure supplement 3—source data 2. [file elife-108438-fig1-figsupp3-data2.zip › Figure 1-figure supplement 3-source data 2/Figure 1-figure supplement 3C (SA-HRP).tif]

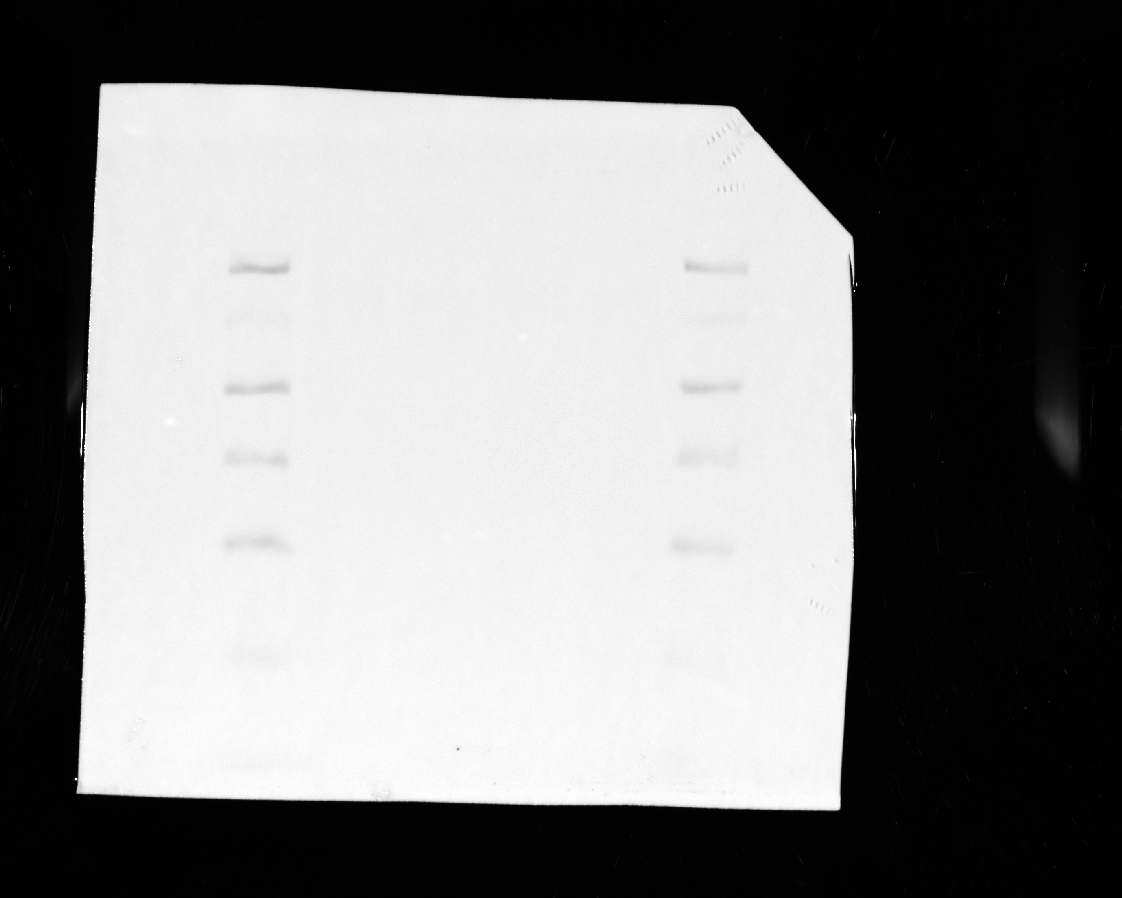

Supplement: Figure 1—figure supplement 3—source data 2. [file elife-108438-fig1-figsupp3-data2.zip › Figure 1-figure supplement 3-source data 2/Figure 1-figure supplement 3D (ladder).tif]

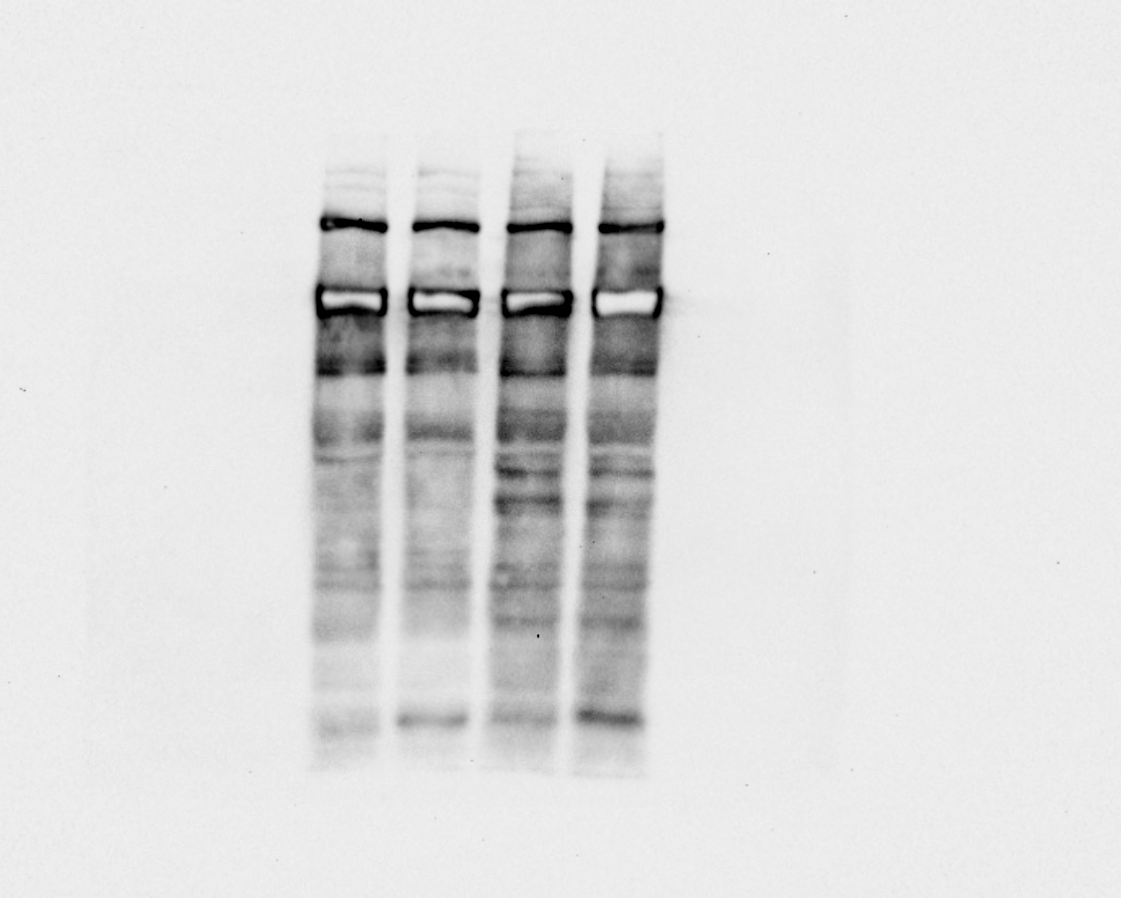

Supplement: Figure 1—figure supplement 3—source data 2. [file elife-108438-fig1-figsupp3-data2.zip › Figure 1-figure supplement 3-source data 2/Figure 1-figure supplement 3D (SA-HRP).tif]

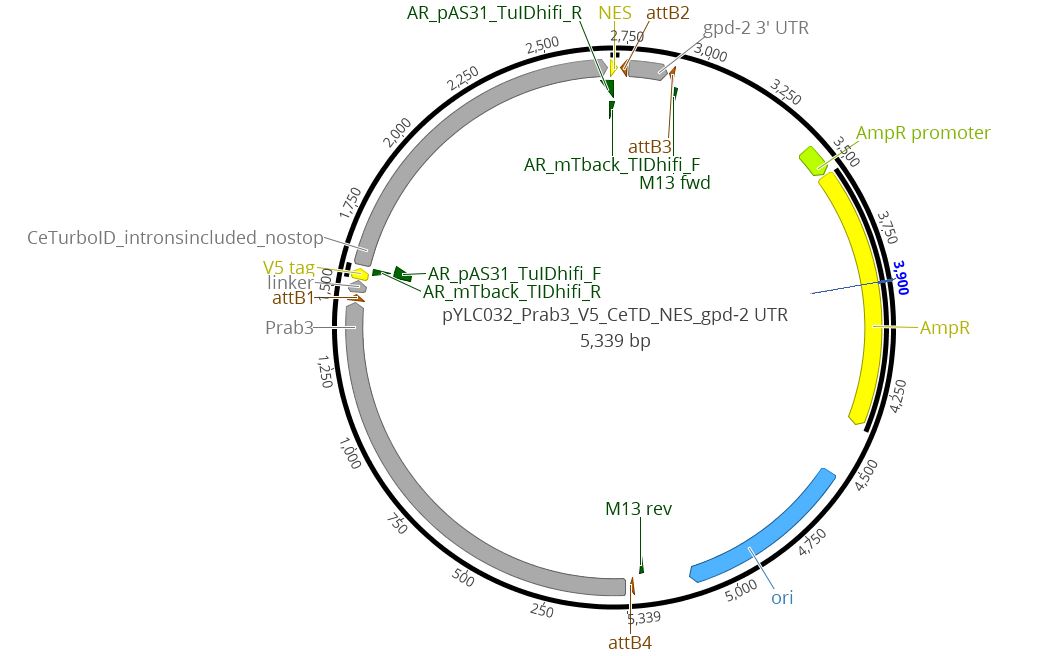

Supplement: Source data 1. — These plasmids encode Prab-1743 3::V5::TurboID::gpd-2 3’ UTR and Prab-3::kin-2(ce179)::SL2::tag-RFP::gpd-2 3’ UTR, 1744 respectively. The folder contains an image of each plasmid map, as well as their DNA sequences 1745 in.fasta and.gb formats. [file elife-108438-data1.zip › Supplementary file 2/pYLC032_Prab3_V5_CeTD_NES_gpd-2 UTR_map.JPG]

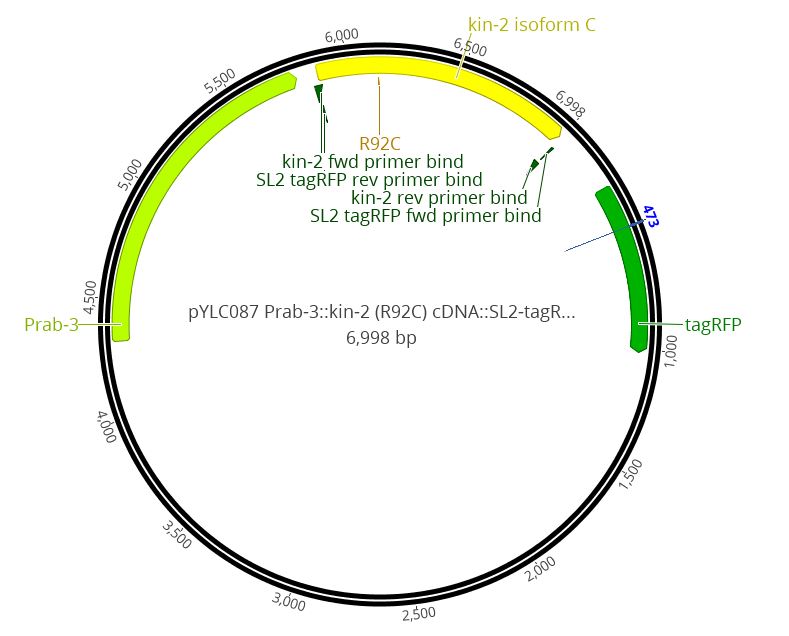

Supplement: Source data 1. — These plasmids encode Prab-1743 3::V5::TurboID::gpd-2 3’ UTR and Prab-3::kin-2(ce179)::SL2::tag-RFP::gpd-2 3’ UTR, 1744 respectively. The folder contains an image of each plasmid map, as well as their DNA sequences 1745 in.fasta and.gb formats. [file elife-108438-data1.zip › Supplementary file 2/pYLC087 Prab-3__kin-2 (R92C) cDNA__SL2-tagRFP_map.JPG]
