# Supplementary material for: Identifying regulators of associative learning using a protein-labelling approach in Caenorhabditis elegans
Source: eLife. 2026 Jan 28;14:RP108438. doi: 10.7554/eLife.108438 (PMC12851583; doi:10.7554/eLife.108438)
Supplement: Figure 1—figure supplement 3—source data 1. [file elife-108438-fig1-figsupp3-data1.zip › Figure 1-figure supplement 3-source data 1/Figure 1-figure supplement 3-source data 1.pdf]

Colorimetric for SA-HRP blot

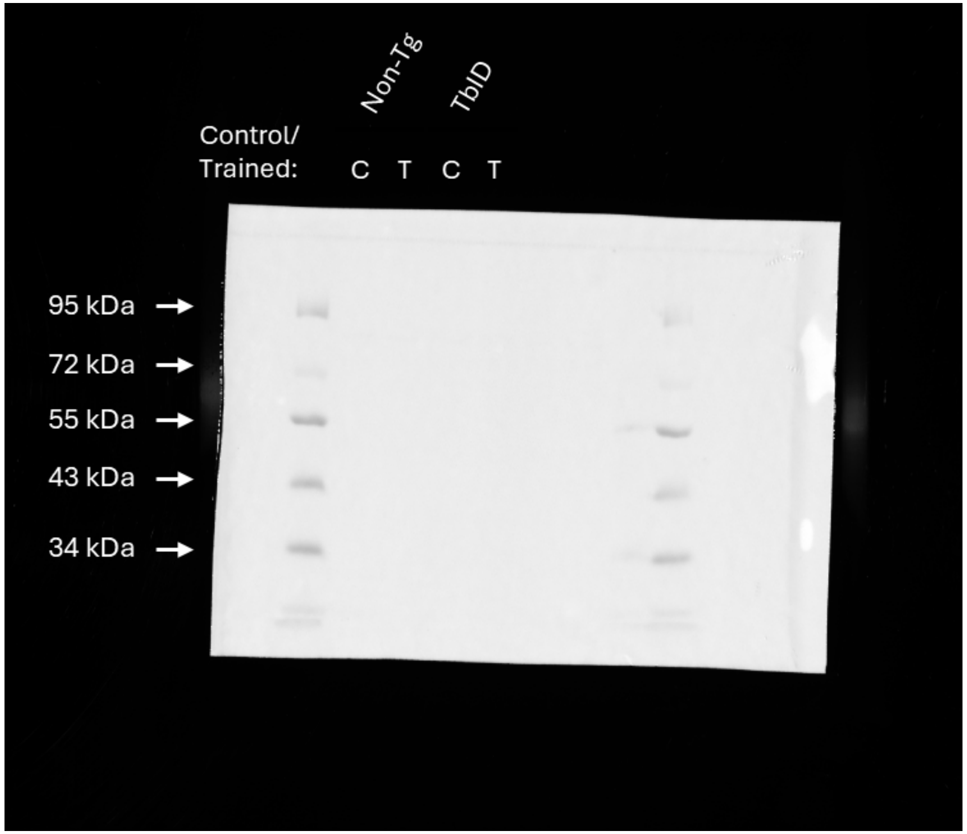

Chemiluminescence for SA-HRP blot

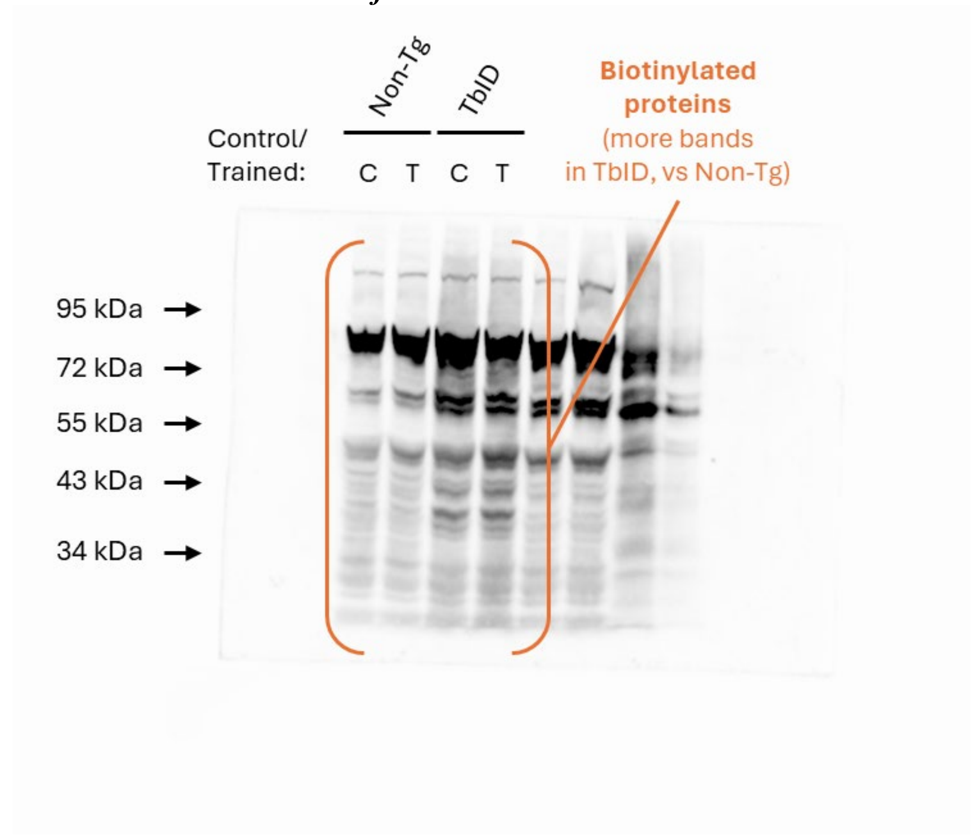

Figure 1 – figure supplement 3A, Source Data 1. Original membrane images corresponding to **Figure 1 – figure supplement 3A**. Lanes contain total protein from biotin-depleted non-transgenic/Non-Tg animals or TurboID/TbID worms undergoing high-salt control (C) or trained (T) treatment in a salt associative learning experiment. *C. elegans* lines and treatment strategies are annotated on the top of each image. Biotinylated proteins were visualised using chemiluminescence via streptavidin-horseradish peroxidase (SA-HRP). Leftmost lane contains protein ladder (shown in the 'colorimetric' image). Molecular weights for protein ladder standards are annotated on the left side of each image (in kDa). Relevant bands are annotated in orange.

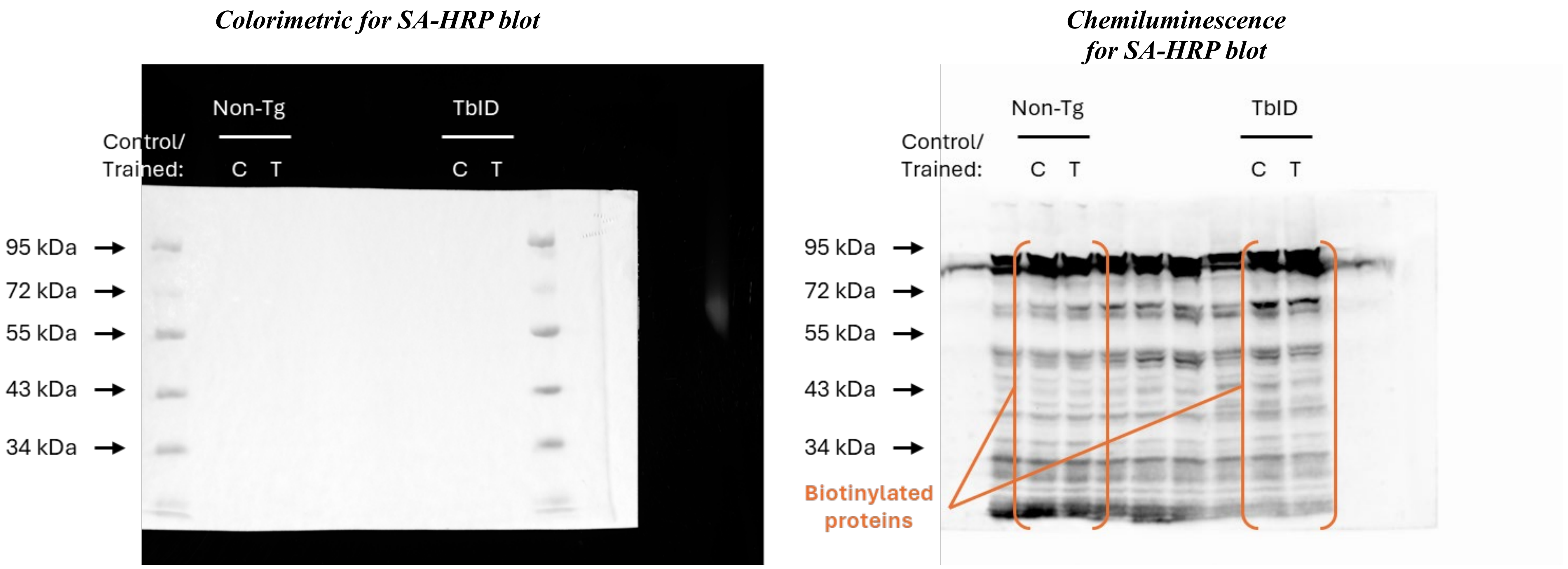

Figure 1 – figure supplement 3B, Source Data 1. Original membrane images corresponding to **Figure 1 – figure supplement 3B**. Lanes contain total protein from biotin-depleted non-transgenic/Non-Tg animals or TurboID/TbID worms undergoing high-salt control (C) or trained (T) treatment in a salt associative learning experiment. *C. elegans* lines and treatment strategies are annotated on the top of each image. Biotinylated proteins were visualised using chemiluminescence via streptavidin-horseradish peroxidase (SA-HRP). Leftmost lane contains protein ladder (shown in the 'colorimetric' image). Molecular weights for protein ladder standards are annotated on the left side of each image (in kDa). Relevant bands are annotated in orange.

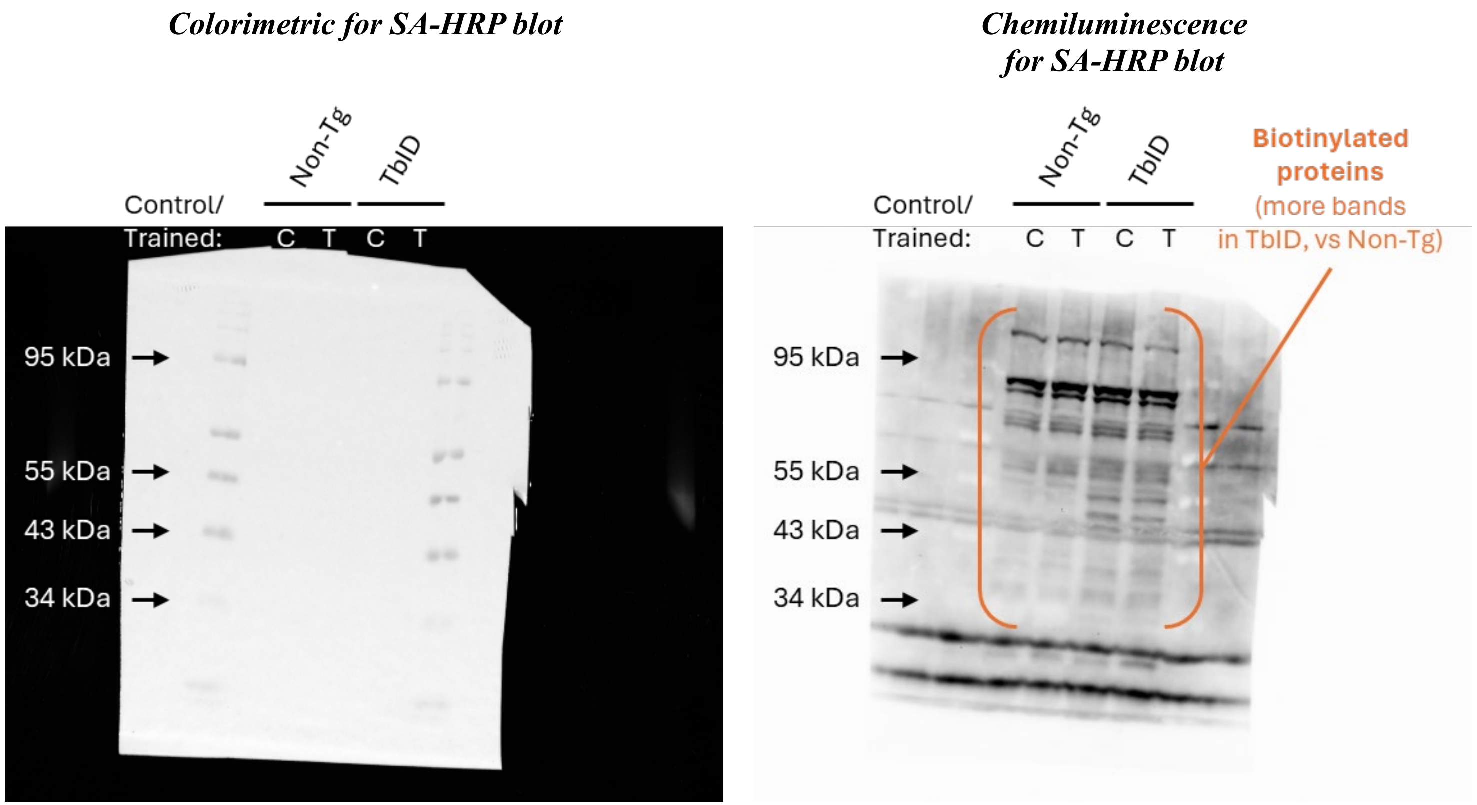

Figure 1 – figure supplement 3C, Source Data 1. Original membrane images corresponding to **Figure 1 – figure supplement 3C**. Lanes contain total protein from biotin-depleted non-transgenic/Non-Tg animals or TurboID/TbID worms undergoing high-salt control (C) or trained (T) treatment in a salt associative learning experiment. *C. elegans* lines and treatment strategies are annotated on the top of each image. Biotinylated proteins were visualised using chemiluminescence via streptavidin-horseradish peroxidase (SA-HRP). Leftmost lane contains protein ladder (shown in the 'colorimetric' image). Molecular weights for protein ladder standards are annotated on the left side of each image (in kDa). Relevant bands are annotated in orange.

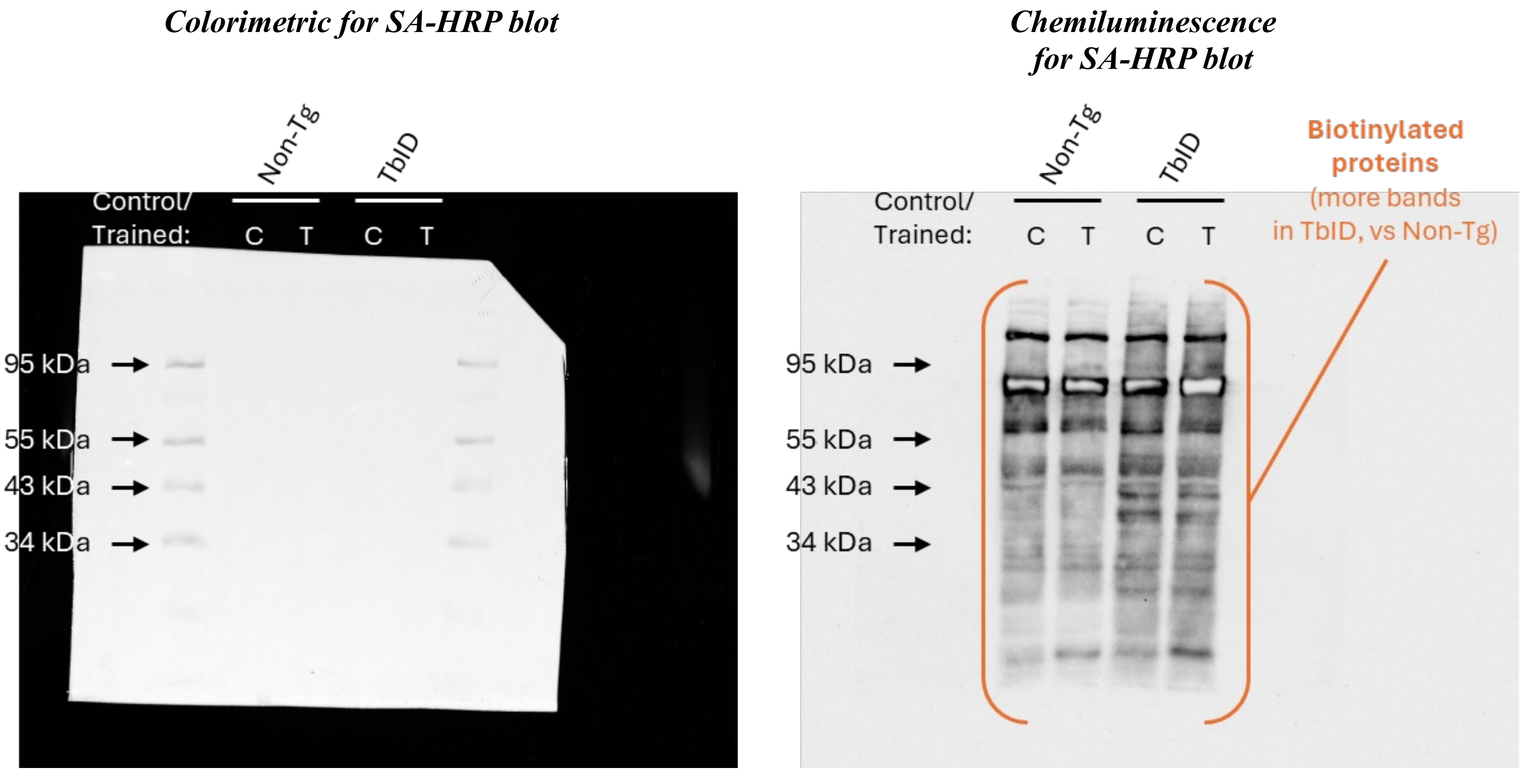

Figure 1 – figure supplement 3D, Source Data 1. Original membrane images corresponding to **Figure 1 – figure supplement 3D**. Lanes contain total protein from biotin-depleted non-transgenic/Non-Tg animals or TurboID/TbID worms undergoing high-salt control (C) or trained (T) treatment in a salt associative learning experiment. *C. elegans* lines and treatment strategies are annotated on the top of each image. Biotinylated proteins were visualised using chemiluminescence via streptavidin-horseradish peroxidase (SA-HRP). Leftmost lane contains protein ladder (shown in the 'colorimetric' image). Molecular weights for protein ladder standards are annotated on the left side of each image (in kDa). Relevant bands are annotated in orange.
